# Dataset for tracing phosphorus sources by the oxygen isotopes of phosphate

**DOI:** 10.1016/j.dib.2022.108717

**Published:** 2022-11-02

**Authors:** Xiuyi Zhao, Rongxiang Tian, Wuhu Feng, Zhan Jin

**Affiliations:** a School of Earth Sciences, Zhejiang University, China; b Key Laboratory of Geoscience Big Data and Deep Resource of Zhejiang Province, China; c National Center for Atmospheric Science, University of Leeds, United Kingdom; d School of Earth and Environment, University of Leeds, United Kingdom; e Guangzhou Research Institute of Environmental Protection, China

**Keywords:** Phosphate source, Oxygen isotopes of phosphate, Atmospheric transport, East China Sea

## Abstract

Phosphorus is an essential nutrient for the growth of marine life. Especially in the East China Sea (ECS) where phosphorus is limited compared to the rest of China's sea, the external input of phosphorus can cause changes in primary productivity, and even induce in harmful algal blooms. In May 2020, the National Natural Science Foundation of China carried out a scientific investigation in the shared Spring Voyage in the ECS. We choose the area between 120.93 ˚ E-125.9 ˚ E and 26.08˚ N - 32.35 ˚ N as research sites for the analysis of the main sources of phosphate in the ECS during summer. The samples were all from the voyage of the research ship (Xiangyanghong 18). Dissolved inorganic phosphorus in seawater was enriched, and dissolved inorganic phosphorus was extracted. Then we measured the oxygen isotopes of phosphate in seawater and introduced the two-component mixing model for the analysis of potential sources of phosphate. In order to further quantitatively analyze the contribution rate of phosphate from different sources in the ECS, we choose a Bayesian isotope mixing model. These data can be used to analyze the contribution of phosphate from different sources in seawater and are helpful to explore the influencing factors of phosphate in the ECS.

**Specifications Table**

**Table:** 

Subject	Oceanography
Specific subject area	Isotopes, phosphate
Type of data	Table, figure
How the data were acquired	Survey (scientific investigation in the National Natural Science Foundation of China Open Research Cruise) (Cruise No. NORC2020-02) in the East China Sea in 19-28 May 2020, funded by the Shiptime Sharing Project of the National Natural Science Foundation of China. This cruise was conducted onboard the R/V “XiangYangHong 18” of The First Institute of Oceanography, Ministry of Natural Resources, China.
Data format	Raw and analyzed
Data source location	City/Town/Region: The East China Sea Country: China
Data accessibility	Repository name: Zenodo Data Data identification number: DOI: 10.5281/zenodo.6568800 Direct URL to data: https://zenodo.org/record/6568800#.YyzQ5tdBw2w

## Value of the Data

• This data represents the oxygen isotope of phosphate in the seawater from the East China Sea (ESC), which can be used to analyze the source of phosphate in the ESC during summer.
• This data can be used to build a model for the quantitative analysis of phosphorus sources using the oxygen isotopes of phosphate
• The East China Sea is a phosphorus-restricted area, but there are few data related to phosphorus. This data can provide a reference for future research on phosphate.
• The data will assist with revealing the occurrence mechanism of harmful algal blooms in the ESC restricted by phosphorus, and have great application value for the effective control of harmful algal blooms.

## 1 Data Description

**Fig. 1:**
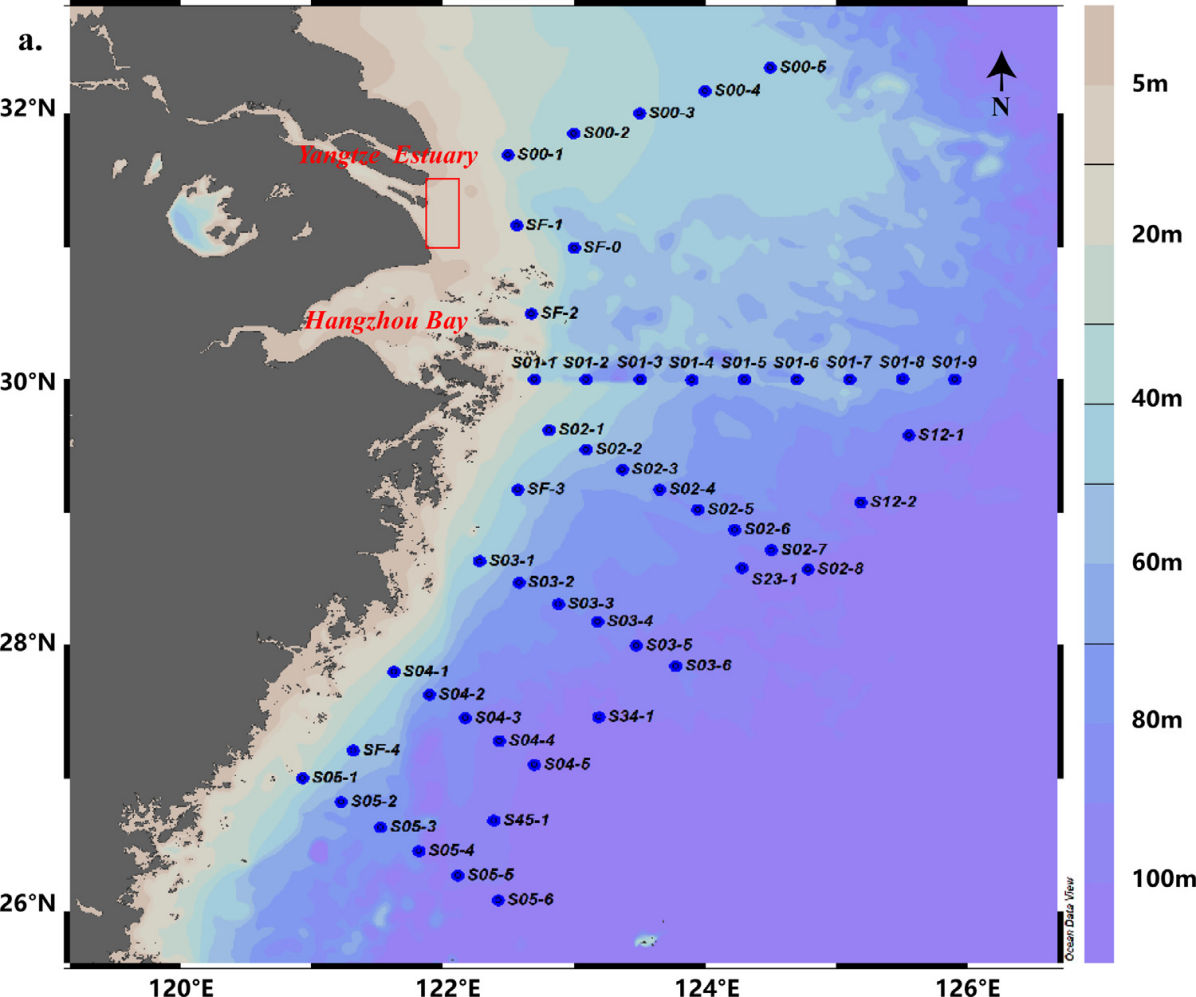
Sampling site. Sampling roadmap for the ESC, 19-28 May 2020, the dots indicate sampling sites, the numbers are serial numbers, and the red rectangle represents the Yangtze Estuary. It is drawn according to software Ocean Data View [1].

This paper contains data for source of phosphate in the ESC in 19-28 May 2020, samples were collected in the shared Spring Voyage. The map of the voyage and station is shown in Fig. 1.

During the voyage, physicochemical parameters (salinity and temperature) and oxygen isotopes of phosphate(δ^18^O_p_) of surface seawater in the ESC can be seen in Table 1. Atmospheric deposition and adjacent sea transportation were used as terrestrial and seawater end-members respectively to construct the δ^18^O_p_ end-member mixing model by calculated the δ^18^O_p_ mixing value [2] (Table 1).

**Table 1:** Physicochemical parameters and δ^18^O_p_ distribution of surface seawater in the ESC from 19 to 28 May 2020.

Station Number	Latitude (N)	Longitude (E)	Temperature (°C)	Salinity (‰)	δ^18^O_p_ (‰)	equilibrium value (‰)	mixing value (‰)
S00-1	31.69	122.50	19.83	28.13	17.37	28.68	terrestrial source
S00-2	31.85	123.00	19.35	29.70	14.01	29.45	16.64
S00-3	32.00	123.50	17.57	31.18	17.46	30.47	15.92
S00-4	32.17	124.00	16.71	31.06	18.33	30.62	15.98
S00-5	32.35	124.50	16.56	31.60	17.10	30.87	15.70
S01-1	30.00	122.70	20.64	29.73	14.66	29.16	16.63
S01-2	30.00	123.10	20.94	30.61	16.68	29.45	16.20
S01-3	30.00	123.51	22.16	32.15	14.08	29.80	15.41
S01-4	30.00	123.90	21.27	31.55	16.04	29.76	15.73
S01-5	30.00	124.30	21.07	32.14	15.19	30.05	15.42
S01-6	30.00	124.70	20.99	31.61	14.92	29.85	15.70
S01-7	30.00	125.10	20.16	32.28	14.89	30.32	15.35
S01-8	30.00	125.50	19.94	31.54	17.02	30.06	15.73
S01-9	30.00	125.90	19.86	31.66	11.04	30.13	15.67
S02-1	29.62	122.81	21.74	29.94	16.24	28.99	16.53
S02-2	29.47	123.09	21.56	30.99	17.18	29.46	16.01
S02-3	29.32	123.37	22.02	32.18	13.67	29.84	15.40
S02-4	29.17	123.65	21.91	33.19	5.10	30.29	14.85
S02-5	29.02	123.94	21.28	32.17	13.03	30.01	15.40
S02-6	28.87	124.22	21.33	32.80	16.09	30.26	15.06
S02-7	28.72	124.50	21.94	33.41	13.29	30.37	14.73
S02-8	28.57	124.78	21.91	33.71	7.60	30.50	14.56
S03-1	28.63	122.28	22.08	30.86	16.18	29.29	16.08
S03-2	28.47	122.58	23.26	33.43	14.76	30.07	14.72
S03-3	28.31	122.88	23.80	33.63	7.63	30.03	14.60
S03-4	28.18	123.18	23.34	33.35	10.10	30.02	14.76
S03-5	28.00	123.48	23.89	33.47	11.88	29.94	14.69
S03-6	27.84	123.78	24.19	34.27	16.63	30.20	14.23
S04-1	27.80	121.63	22.92	31.34	15.52	29.29	15.84
S04-2	27.63	121.90	25.05	34.22	12.37	29.98	14.26
S04-3	27.45	122.17	24.79	34.34	16.26	30.09	14.19
S04-4	27.28	122.43	26.04	34.46	14.12	29.85	seawater source
S04-5	27.10	122.70	24.29	34.09	17.21	30.10	14.34
S05-1	27.00	120.93	23.08	31.86	16.61	29.47	15.57
S05-2	26.82	121.23	24.49	34.33	18.12	30.15	14.20
S05-3	26.63	121.53	24.83	34.39	15.50	30.10	14.16
S05-4	26.45	121.82	24.71	34.44	17.55	30.15	14.13
S05-5	26.27	122.12	25.12	34.37	17.96	30.03	14.17
S05-6	26.08	122.42	23.68	34.45	17.35	30.39	14.13
S12-1	29.58	125.55	20.28	33.38	17.81	30.74	14.74
S12-2	29.07	125.18	21.30	33.25	16.86	30.45	14.82
S23-1	28.58	124.28	21.64	32.63	16.17	30.12	15.16
S34-1	27.46	123.19	24.19	34.29	18.65	30.21	14.22
S45-1	26.68	122.39	24.81	34.31	15.26	30.08	14.21
SF-0	30.99	123.00	15.70	31.39	16.76	30.99	15.81
SF-1	31.16	122.56	20.23	29.16	17.21	29.02	16.90
SF-2	30.49	122.68	19.94	30.70	18.27	29.72	16.16
SF-3	29.17	122.57	21.80	30.10	17.47	29.04	16.46
SF-4	27.21	121.32	23.28	32.20	16.46	29.56	15.39

**Fig. 2:**
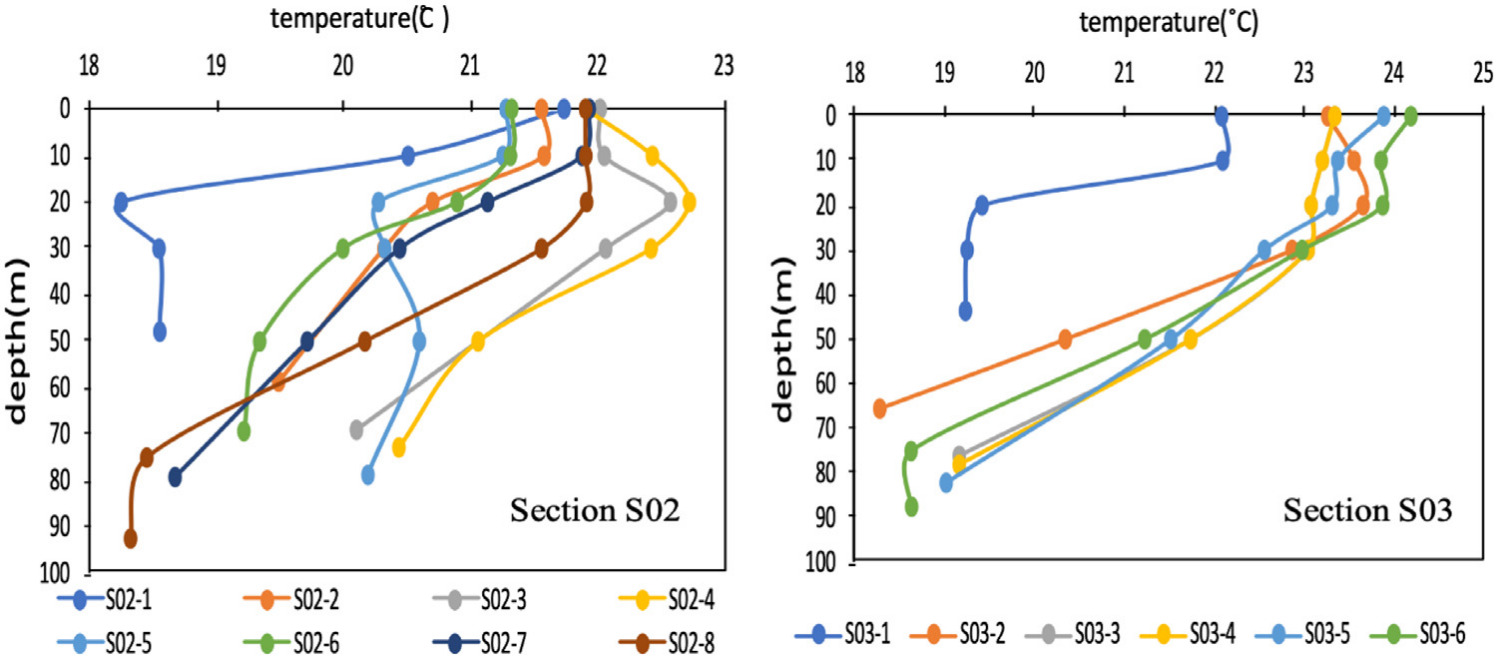
Temperature variation with depth at each station on the S02 (left) and S03 (right) sections.

**Fig. 3:**
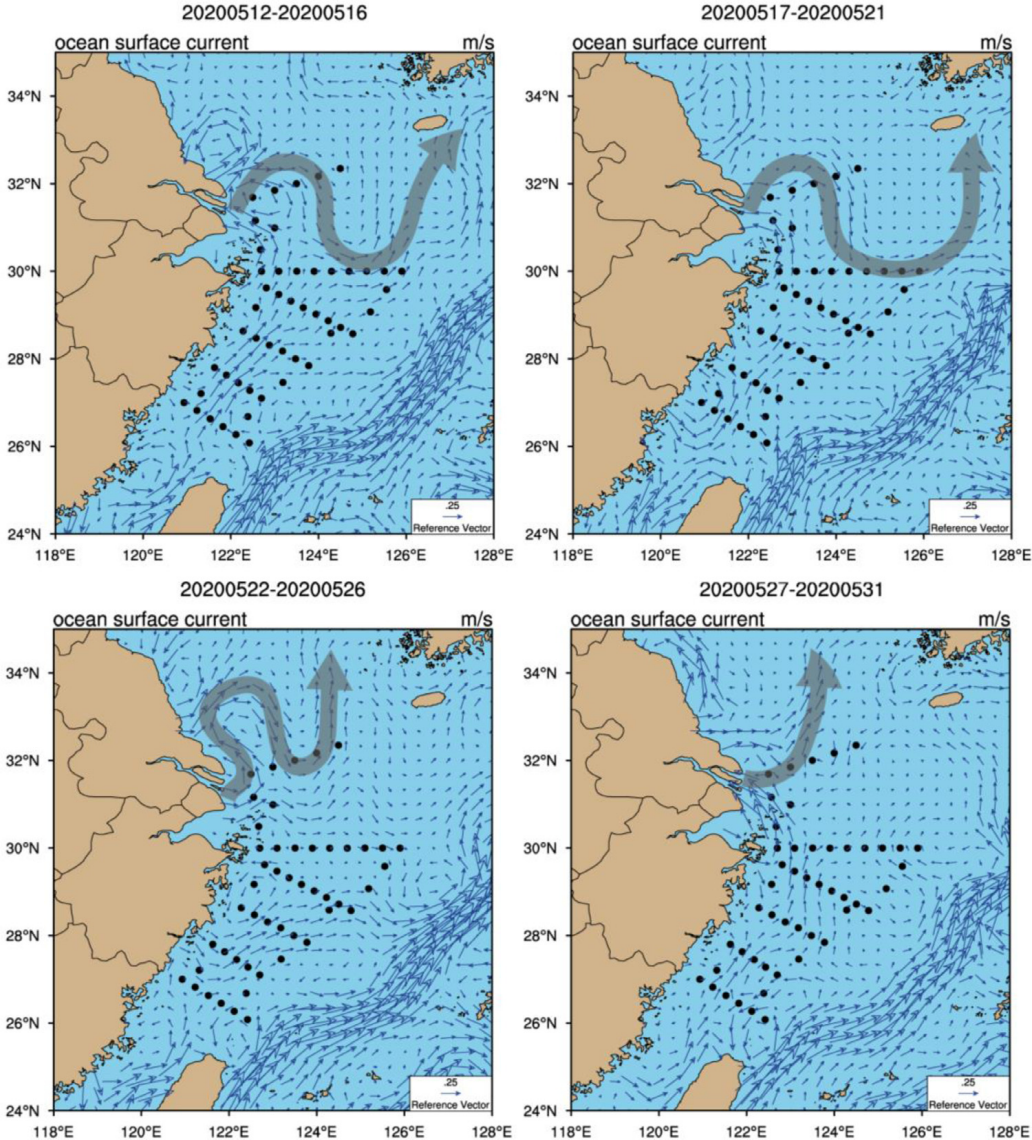
Surface water currents in the ESC during voyage 12-31 May 2020, grey arrows indicate the main direction of ocean currents off the Yangtze River Estuary.

**Fig. 4:**
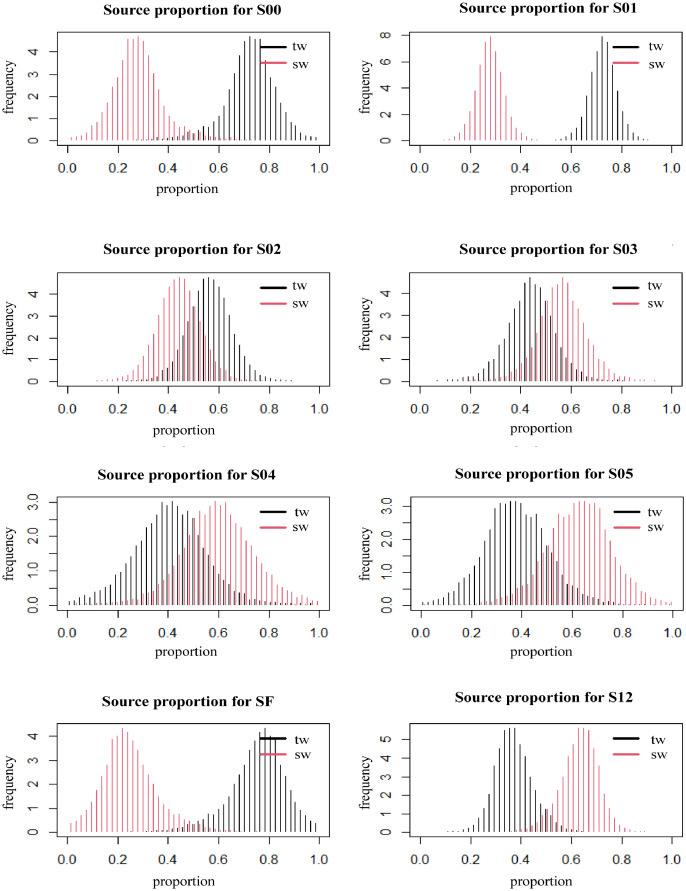
Contribution of terrestrial sources and seawater sources at different sections in the ESC (tw: terrestrial source, sw: seawater source).

It can be seen from the changes of temperature with depth at each station (Fig. 2). For Yangtze River diluted water, we draw a map of surface water currents by data from https://search.earthdata.nasa.gov (Fig. 3). The Stable Isotope Analysis in R (SIAR) model [3] was used to analyze the contribution rate of the sources to phosphate during navigation (Fig. 4).

**Fig. 5:**
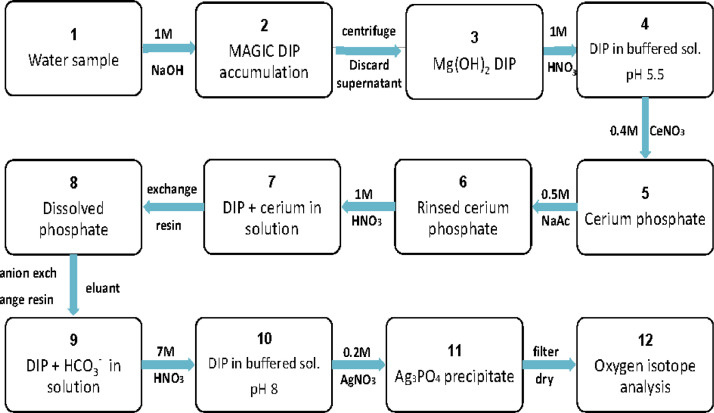
Flow chart of enrichment and extraction of dissolved inorganic phosphorus in seawater. The design of the flow chart refers to (4-6).

## 2 Experimental Design, Materials and Methods

(1) Sample collection and analysis

Surface seawater was collected at 49 stations for δ^18^O_p_ analysis by conductivity-temperature-depth profiler from the voyage of the research ship (Fig. 1). Magnesium-induced coprecipitation (MAGIC) was used to enrich dissolved inorganic phosphorus in seawater [4,5], and the dissolved inorganic phosphorus was extracted by the method of Tcaci [6]. The δ^18^O_p_ of the sample was determined by a high-temperature split-stable isotope mass spectrometer (Flash EA 1112 HT-MAT253, Thermo) and repeated at 10-sample intervals.

In order to ensure the accuracy and stability of the measurement results for each sample, a standard silver phosphate sample was measured for calibration after every ten samples were measured. The original phosphate oxygen isotope values were calculated by Isodat software and then corrected with standard silver phosphate.

(2) Data analysis method

The δ^18^O_p_ values in seawater are affected by water masses with different phosphate concentrations [7]. McLaughlin [2] introduced the two-component mixing model for the analysis of water masses that control phosphate *and calculated the δ^18^O_p_ mixing value. The formula is as follows:*

(1) $$f_{a}+f_{b}=1$$

(2) $$f_{a}S_{a}+f_{b}S_{b}=S_{m}$$

(3) $$\delta_{m}=\frac{\left[\left(f_{a}\times C_{a}\times \delta_{a}\right)+\left(1-f_{a}\right)\times C_{b}\times \delta_{b}\right]}{\left[\left(f_{a}\times C_{a}\right)+\left(1-f_{a}\right)\times C_{b}\right]}$$

Where $f_{a}$, $f_{b}$ represent the proportion of freshwater and seawater respectively, $S_{a}$, $S_{b}$ represent the salinity of two sources, respectively, $S_{m}$ represents the salinity of the research station, $C_{a}$, $C_{b}$ individually represent the phosphate concentration of both sources, $\delta_{a}$, $\delta_{b}$ respectively represent their δ^18^O_p_ value, and $\delta_{m}$ is the calculated δ^18^O_p_ value of the mixing.

If the phosphate is fully utilized by living organisms, the δ^18^O_p_ reaches the theoretical equilibrium value, which can be calculated by the following formula:

(4) $$\delta^{18}O_{Pe}=\left(0.142\times S-4.20\right)-\left[\frac{T-111.4}{4.3}\right]$$

*where δ^18^O_pe_ represents the theoretical equilibrium value, S represents the salinity of the research station, and T represents the temperature of the research station.*

We use this model to identify source of phosphate in the ECS. To quantify the source of phosphate in the ECS, a Bayesian isotope mixing model run in the R software package (Stable Isotope Analysis in R, SIAR) was selected for analysis [3]. It is assumed that there are two main sources. The mean value and standard deviation of oxygen isotope values from different sources are calculated and then put these values into model. Finally, the contribution rate of different sources to phosphate during navigation was used to draw the conclusion (Fig. 4).

All data are underway, and they have been placed in Zenodo repository [8].

## CRediT Author Statement

**Zhao Xiuyi:** Sample collection, Analysis, Software; **Tian Rongxiang:** Design of research scheme; **Zhan Jin:** Analysis; **Wuhu Feng:** Revised the manuscript; **Ran Xiangbin** and **Liu Jun:** Sample collection and Analysis.

## Declaration of Competing Interest

The authors declare that they have no known competing financial interests or personal relationships that could have appeared to influence the work reported in this paper.

## Data Availability

Contribution of atmospheric transport to phosphorus in the East China Sea in summer (Original data).
